# Management of malignant carinal involvement using ‘side‐by‐side’ method of bilateral self‐expandable metallic stents

**DOI:** 10.1002/rcr2.396

**Published:** 2018-12-10

**Authors:** Takayuki Takeda, Hideki Itano, Ryo Wakasa, Masahiko Saitoh, Sorou Takeda

**Affiliations:** ^1^ Department of Respiratory Medicine Uji‐Tokushukai Medical Center Kyoto Japan; ^2^ Department of Thoracic Surgery Uji‐Tokushukai Medical Center Kyoto Japan

**Keywords:** Bilateral self‐expandable metallic stents, central airway obstruction, interventional pulmonology, malignant main carinal involvement, side‐by‐side method

## Abstract

Central airway obstruction caused by cancer requires urgent interventional pulmonology. Malignant main carinal involvement is one of the most challenging situations, usually treated by rigid bronchoscopic intervention under general anaesthesia. However, these patients tend to be in poor condition due to underlying malignancy. Therefore, less‐invasive strategies are needed. Six patients with lung cancer exhibiting malignant carinal involvement treated using the ‘side‐by‐side’ method of bilateral self‐expandable metallic stents using fibre‐optic bronchoscopy under topical anaesthesia were retrospectively investigated. The median procedure time was 29.5 min (range: 23–38), and the palliation of dyspnoea was achieved in all cases. The median survival time after intervention was 58.5 days (range: 23–172). The cause of death was acute coronary syndrome in two patients, aspiration pneumonia in two, upper gastrointestinal perforation in one, and catheter‐related blood stream infection in one. This strategy was less invasive and suitable for patients with poor performance status.

## Introduction

Central airway obstruction (CAO) is critical and life‐threatening for cancer patients and is categorized as an oncological emergency that requires urgent intervention. Therapeutic bronchoscopy using mechanical debridement, brachytherapy, tumour ablation, or airway stent placement in inoperable lung cancer patients with symptomatic airway obstruction is recommended by the third edition of American College of Chest Physicians Evidence‐Based Clinical Practice Guidelines 1. Rigid bronchoscopy plays an invaluable role in the management of CAO, which usually involves several risks factors. Management techniques for CAO are complex and require the total comprehension of complicated and specific strategies dependent on the status of the patient 2.

Malignant carinal involvement is considered one of the most difficult situations among CAO, which is usually treated by rigid bronchoscopic intervention under general anaesthesia. Laser treatment and mechanical debulking are usually applied to remove the tumour invasion, which are followed by Y stent placement to prevent tumour regrowth and strengthen airway patency 3, 4.

The Dumon stent has been the most prevalent among silicone stents 5. The Dumon Y‐stent following tumour debulking is suitable for malignant carinal involvement, which is easily adjusted on a case‐by‐case basis, easily repositioned, and removed if necessary 3. Silicone stents need a special applicator through rigid bronchoscopy, and post‐procedure sputum retention requires scheduled bronchial toileting by fibre‐optic bronchoscopy (FOB).

Patients with malignant carinal involvement tend to have a poor performance status (PS) due to disease progression; consequently, silicone stenting is not applicable in these patients. Thus, less‐invasive interventional strategies are urgently required.

Self‐expandable metallic stents (SEMSs) have an outstanding advantage, namely, their convenient delivery using FOB under topical anaesthesia. Their conformability to the airway structure due to their self‐expansive characteristics is another advantage. On the other hand, the additional therapy to tumour ingrowth or overgrowth is limited, and SEMSs cannot be removed when they become unnecessary.

The application of ‘side‐by‐side’ method of bilateral SEMSs has been reported in the management of malignant carinal involvement 6, 7. This technique is useful in the treatment of patients with poor PS who would not tolerate general anaesthesia.

We retrospectively analysed six patients who were treated using this method in terms of effectiveness, tolerance, and safety of the procedure.

## Case Series

### Patients and Method

#### *Patients*

A retrospective study was performed of patients with lung cancer who developed malignant carinal involvement and were treated using the ‘side‐by‐side’ method of bilateral SEMSs between 2013 and 2017.

The indication criteria for bilateral SEMSs at our hospital are as follows: the airway stenosis is too severe for radiotherapy, which induces oedema of bronchial mucosa at the site of stenosis leading to the total occlusion in either of the mainstem bronchus; histology of non‐small‐cell lung cancer as its prognosis in response to chemotherapy is less encouraging compared to small cell lung cancer; and the rigid bronchoscopic intervention under general anaesthesia is not suitable due to poor PS or complications as decided by both anaesthesiologists and thoracic surgeons.

The patients were numbered sequentially from 1 to 6, and individual data were managed according to the allocated number.

#### *Procedure of the bilateral SEMSs*

**Figure 1:**
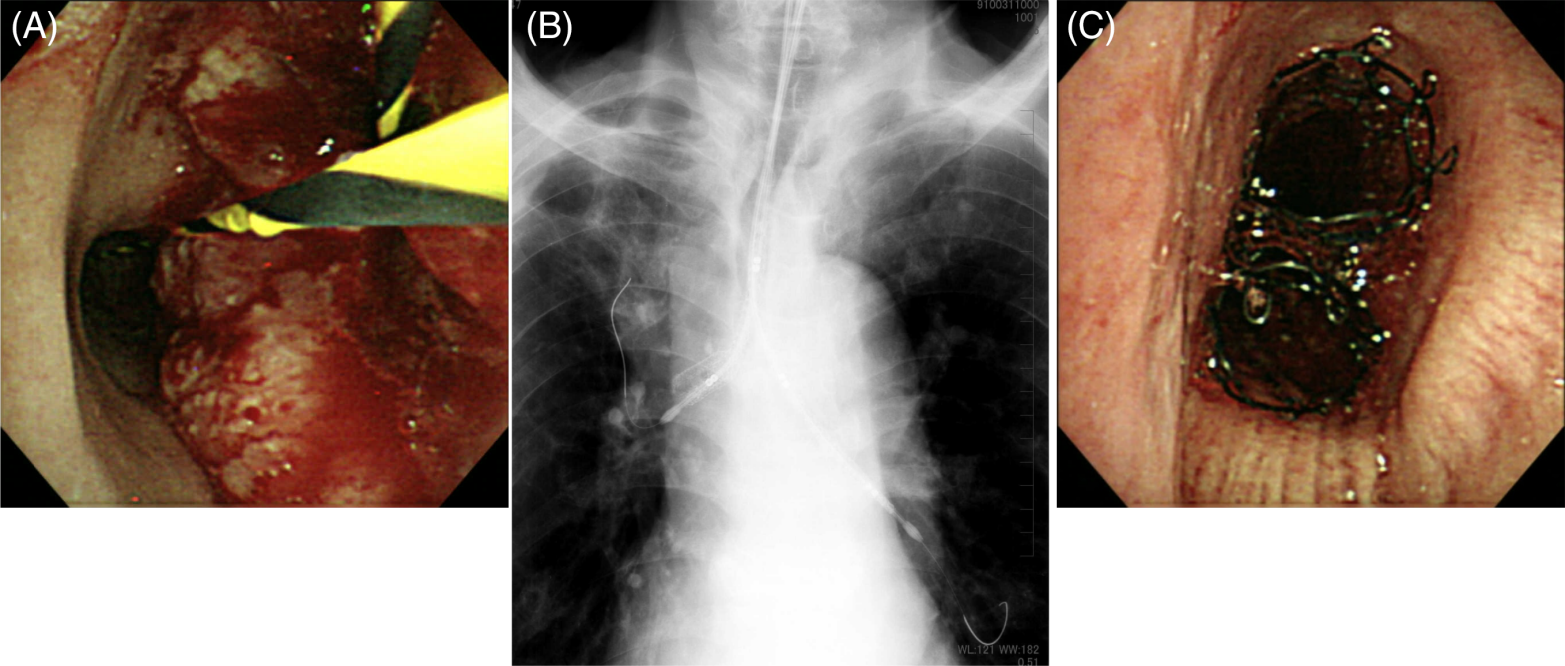
The entire procedure of ‘side‐by‐side’ method in detail (case no.2). A guidewire was inserted into the right mainstem bronchus (RMB), and a similar guidewire was subsequently inserted into the left (A). Then, an Ultraflex‐covered stent mounted on a delivery catheter was advanced to the RMB under fluoroscopic guidance. A similar stent was immediately delivered to the left mainstem bronchus, adjusting the proximal end to the right counterpart (B). After the full expansion of the right stent, the left stent was also released without delay, adjusting the proximal ends (C).

A fibre‐optic bronchoscope was inserted after delivering 4% lidocaine by nebulizer, which was followed by an intravenous administration of midazolam at a dose of 0.06–0.1 mg/kg. Skin markers were placed at the level of carina and 3 cm rostral site. As the most critical sites were in the right mainstem bronchus (RMB) in all cases, a flexible 0.035‐in. guidewire (Jagwire super‐stiff, Boston Scientific Corporation, Marlborough, MA, USA) was inserted into the RMB, and a similar guidewire was subsequently inserted into the left (Fig. 1A). Then, an Ultraflex‐covered stent (14 mm in diameter, 6 cm in length, distal release, Boston Scientific Corporation) mounted on a delivery catheter was advanced to the RMB under fluoroscopic guidance, adjusting the proximal end to the rostral marker. A similar stent was immediately delivered to the left mainstem bronchus, adjusting the proximal end to the right counterpart (Fig. 1B). The right stent was carefully released, and the full expansion was confirmed; then, the left stent was also released without delay, adjusting the proximal ends. After the procedure, the proximal ends of both stents were tuned using a forceps (Fig. 1C).

Blood pressure was monitored every 2 min, and SpO_2_ was monitored in real time. Oxygen administration rate during the procedure was set at 2 L/min more than the baseline.

#### *Calculation of the time needed for the bilateral SEMSs*

The entire time needed was calculated from the first insertion of FOB to the removal of FOB after the final check of the expansion of SEMSs.

#### *Outcomes of the procedure in terms of subjective assessments, stent patency, and post‐procedure survival*

Subjective symptoms were monitored before and after the procedure and were quantitatively assessed by the modified Medical Research Council (mMRC) dyspnoea grade 8. The treatment was evaluated as efficient when the mMRC dyspnoea grade improved by 1 point.

Stent patency and post‐procedure survival, as well as the cause of death, were collected from the medical records.

#### *Statistical analysis*

Data are presented as median (range) for continuous variables.

## Results

### Baseline Patient Characteristics

The median age was 70.0 years (range: 66–91); one patient was female, and five were male. Two were former smokers, and four were current smokers. The histology was squamous cell carcinoma in four and adenocarcinoma without oncogenic driver mutation in two. The primary sites were right upper lobe in five patients and mediastinum in one. SEMSs had been placed in RMB before the procedure in two patients; then, the ‘stent in stent’ technique was utilized. The baseline mMRC dyspnoea grades were: grade 3, *n* = 3; grade 4, *n* = 3.

### Time Needed for the Procedure and Complications

The median time needed for the entire procedure was 29.5 min (range: 23–38). There were no complications, including uncontrollable bleeding, bronchial perforation, pneumothorax, and respiratory failure.

### Computed tomography Images of the ‘Side‐by‐Side’ Method

**Figure 2:**
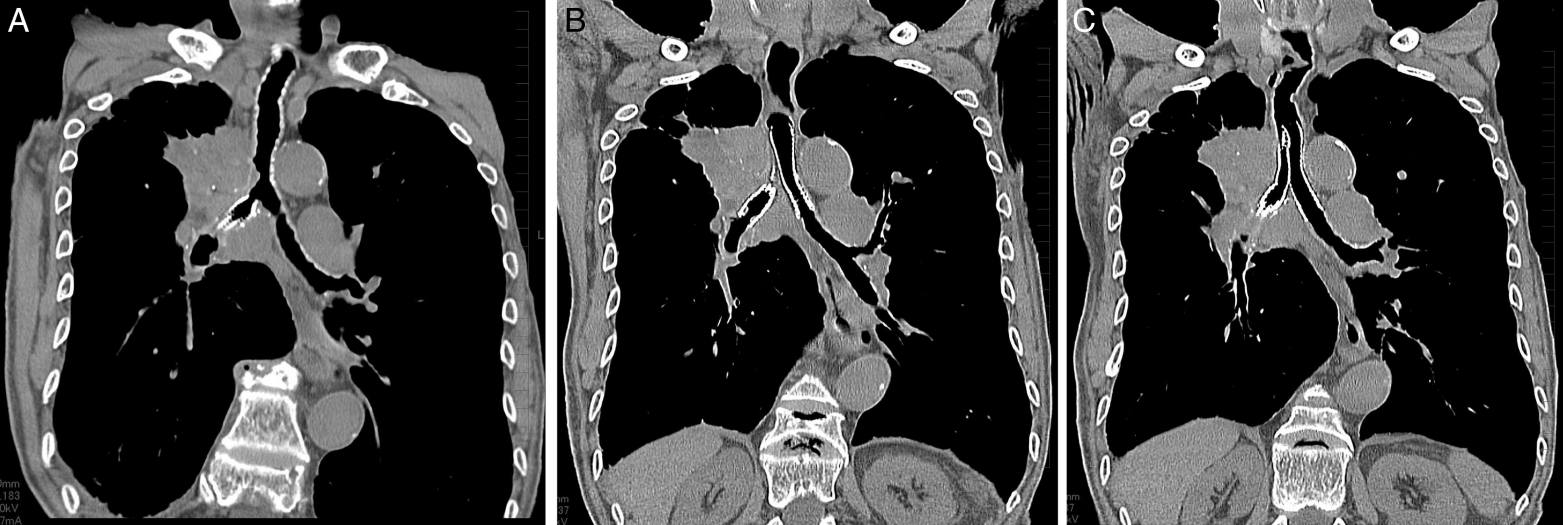
The representative chest coronal computed tomography images are shown before (A) and 7 days after (B, C) the ‘side‐by‐side’ method of bilateral self‐expandable metallic stents (SEMSs) in case no.2. Severe stenosis was observed in the right mainstem bronchus, which had already been treated with an self‐expandable metallic stent (SEMS) as well as the lower trachea and the orifice of the left mainstem bronchus (A). Bilateral SEMSs were patent and adjacent to each other (B, C), and each of the proximal end was in line with each other without migration (C) 14 days after the procedure.

The representative chest coronal computed tomography (CT) images are shown in Figure 2, before (A) and 7 days after (B, C) the ‘side‐by‐side’ method in case no. 2.

**Figure 3:**
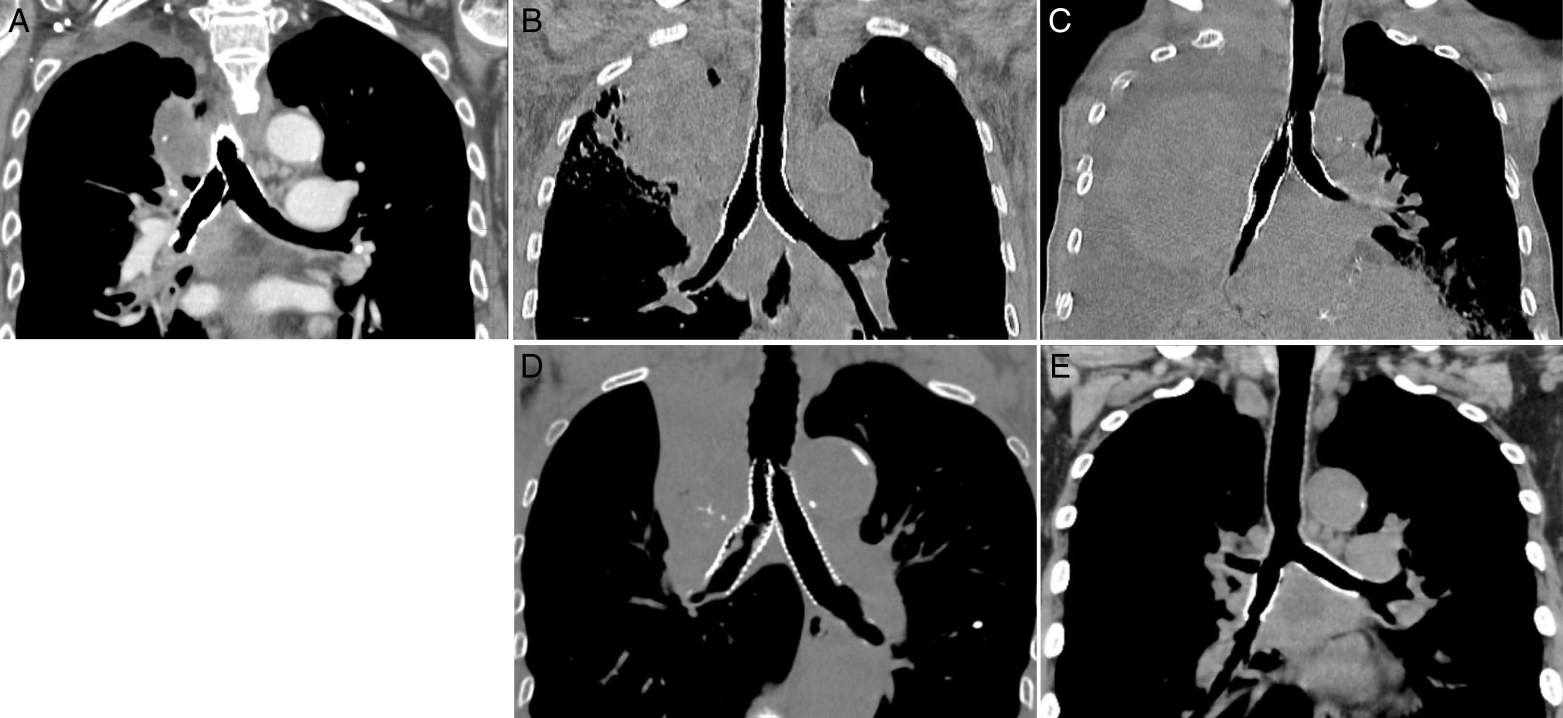
The multi‐planar reconstruction coronal images of chest computed tomography images after the procedure in cases no.1 and 3–6 (A, B, C, D, and E, respectively). The stents were all patent in five patients, while the initial expansion of the right lung by the procedure ended in a total occlusion by tumour overgrowth at the distal site of the right stent after 14 days due to rapid progression of the main tumour (C).

The chest coronal CT images after the procedure in cases 1 and 3–6 are shown in Figure 3.

### The Outcomes of the Procedure (Subjective Assessment, Stent Patency, Post‐Procedure Survival, and the Cause of Death)

Subjective improvement in dyspnoea was observed in all cases. The mMRC dyspnoea grades were improved by −2 grades in three patients and −1 grade in three.

The stent patency was confirmed at the time of death in five cases, while the occlusion was observed 14 days after the procedure in one case. The median time of stent patency and post‐procedure survival were 58.5 days (range: 14–172) and 58.5 days (range: 23–172), respectively.

The causes of death were acute coronary syndrome in two patients, aspiration pneumonia in two, catheter‐related blood stream infection in one, and upper gastrointestinal perforation in one.

### Post‐Treatment after the Procedure

Two patients chose bilateral SEMSs as best supportive care; thus, they did not receive any additional treatment. Four patients were treated with one regimen of cytotoxic chemotherapy; however, all of them refused further chemotherapy, and all of them died after the completion of the relevant chemotherapy. The chemotherapy regimens were as follows: carboplatin plus paclitaxel, carboplatin plus gemcitabine, carboplatin plus S‐1, and docetaxel plus ramucirumab in each.

## Discussion

The application of the ‘side‐by‐side’ method of bilateral SEMSs to malignant carinal involvement was less invasive and safe under topical anaesthesia in all cases.

Although rigid bronchoscopy is recommended and plays an important role in the management of CAO 1, 2, the ‘side‐by‐side’ method could be suitable in cases unfit for rigid bronchoscopic intervention due to poor PS or complications.

In terms of the palliation of suffocating symptoms, this procedure was quite effective in all cases, with a prompt improvement in mMRC dyspnoea grade. As the prognosis of patients suffering from malignant carinal involvement is poor, the palliation of symptoms should be considered important as a short‐term clinical benefit.

On the other hand, stent patency and post‐procedure survival are also important as long‐term clinical benefits. The median time of stent patency and post‐procedure survival could be long enough considering the critical status at the time of bilateral SEMSs placement. Five patients demonstrated stent patency at the time of death, except one with stent occlusion after 14 days due to disease progression. Furthermore, the cause of death in six patients was not due to stent‐related issues but because of other causes irrelevant to the tumour progression. Thus, this procedure could be a strong strategy in the management of malignant carinal involvement with poor PS. Post‐procedure treatment with chemotherapy and general management including ischaemic heart disease are also crucial in the pursuit of longer overall survival.

The median time of stent patency after the ‘side‐by‐side’ method was 58.5 days, while that after the Dumon Y‐stent was reported to be 133 days 3. The shorter stent patency after the ‘side‐by‐side’ method compared with Dumon Y‐stent could be due to the poorer baseline PS unfit for rigid bronchoscopy.

In an attempt using the less‐invasive strategies, a combination of two types of metallic stents 9 and the development of new windowed stents 10 have already been reported. In the five‐case series of two types of metallic stent, the median post‐procedure survival time was 35 days (range: 7–140), and the cause of death was due to disease progression in three, pneumonia in one, and respiratory failure in one 9. In the six‐case series of windowed stents, the average time of stent patency was 159 days, which could be due to their good design conforming to the bifurcation 10. As these methods requires specific metallic stents and are unfit for the emergent use with longer time needed, we used the pre‐existing SEMSs.

The current study has several limitations. First, as this study was a retrospective observational investigation with a small sample size, the feasibility should be elucidated in prospective studies with larger populations. However, this could be difficult as malignant carinal involvement is one of the specific and rare forms of CAO. Thus, it is important to consider the efficacy as well as the risk and invasiveness of this method compared to Dumon Y‐stent according to the background of each case. The discussion by respiratory physicians, thoracic surgeons, radiotherapists, and anaesthesiologists is indispensable in such cases.

In conclusion, the management of malignant carinal involvement using the ‘side‐by‐side’ method of bilateral SEMSs was effective and tolerable without any complication in cases of poor PS or with cardiovascular complications.

### Disclosure Statement

This study was carried out in accordance with the Declaration of Helsinki and was approved by the Ethics Committee of Uji‐Tokushukai Medical Center (approval date: 17 July 2018; approval number: 2018‐08). Written informed consent was omitted as the study was retrospective, and patient anonymity was assured.
